# Genetic engineering of marine cyanophages reveals integration but not lysogeny in T7-like cyanophages

**DOI:** 10.1038/s41396-021-01085-8

**Published:** 2021-08-24

**Authors:** Dror Shitrit, Thomas Hackl, Raphael Laurenceau, Nicolas Raho, Michael C. G. Carlson, Gazalah Sabehi, Daniel A. Schwartz, Sallie W. Chisholm, Debbie Lindell

**Affiliations:** 1 Faculty of Biology, Technion—Israel Institute of Technology, Haifa, Israel; 2 Department of Civil and Environmental Engineering, Department of Biology, Massachusetts Institute of Technology, Cambridge, MA USA

**Keywords:** Microbial ecology, Bacteriophages

## Abstract

Marine cyanobacteria of the genera *Synechococcus* and *Prochlorococcus* are the most abundant photosynthetic organisms on earth, spanning vast regions of the oceans and contributing significantly to global primary production. Their viruses (cyanophages) greatly influence cyanobacterial ecology and evolution. Although many cyanophage genomes have been sequenced, insight into the functional role of cyanophage genes is limited by the lack of a cyanophage genetic engineering system. Here, we describe a simple, generalizable method for genetic engineering of cyanophages from multiple families, that we named REEP for REcombination, Enrichment and PCR screening. This method enables direct investigation of key cyanophage genes, and its simplicity makes it adaptable to other ecologically relevant host-virus systems. T7-like cyanophages often carry integrase genes and attachment sites, yet exhibit lytic infection dynamics. Here, using REEP, we investigated their ability to integrate and maintain a lysogenic life cycle. We found that these cyanophages integrate into the host genome and that the integrase and attachment site are required for integration. However, stable lysogens did not form. The frequency of integration was found to be low in both lab cultures and the oceans. These findings suggest that T7-like cyanophage integration is transient and is not part of a classical lysogenic cycle.

## Introduction

Marine cyanobacteria of the genera *Synechococcus* and *Prochlorococcus* are abundant primary producers that span vast areas of the oceans [1]. Their viruses (cyanophages) are also highly abundant [2–4] and influence cyanobacterial ecology and evolution [5–7], as well as global biogeochemical cycles [8–10]. All currently known cyanophages are tailed, double-stranded DNA viruses belonging to the *Myoviridae*, *Podoviridae* and *Siphoviridae* families [3, 11–15]. Over the last two decades, dozens of cyanophage genomes from isolates and metagenomes have been sequenced [13–21]. Analyses of these genomes have contributed insights into the general properties and lifestyle of marine cyanophages. For example, the T4-like cyanophages from the *Myoviridae* family and the T7-like cyanophages from the *Podoviridae* family share sets of core genes with the *Escherichia coli* T4 and T7 phages, respectively [13, 15, 18, 22]. These genes are involved in virion formation, DNA replication and packaging, transcription and other fundamental processes of the lytic infection cycle. In addition, cyanophage genomes contain many intriguing and unusual features. In particular, they encode a variety of “auxiliary metabolic genes” (AMGs) [10, 14, 22–25] of (cyano)bacterial origin, including those linked to photosynthesis [26, 27], carbon metabolism [28] and nutrient utilization [18, 29], as determined from homology-derived functional assignments. Furthermore, a large reservoir of diverse genes with no putative function is present in cyanophage genomes [16–18, 22], as found for other viruses in the environment [30]. Some of these genes are conserved in multiple cyanophage types [16, 18, 21, 22] whereas others have no database match, and are termed “ORFans” [30, 31].

Elucidating cyanophage gene functions and their role in infection is key for understanding their ecological and evolutionary interactions with their cyanobacterial hosts. Heterologous expression experiments [32–34], in-vitro biochemical assays [32, 35] and comparisons between phage strains with different gene contents [28, 36] have shed light on the function of some cyanophage genes. However, to elucidate the role of these genes in the infection process and to determine their contribution to cyanophage fitness and evolution it is necessary to generate phage mutants and directly compare infection between phages with and without the gene of interest. Thus, a genetic engineering system that is simple and easily transferable between host-virus systems will provide a powerful tool for investigating the function of a wide range of genes in a suite of ecologically relevant cyanophage-cyanobacterial systems.

Lysogeny is a well-known phenomenon in which a virus remains in a non-lytic state within the host, typically after integrating its DNA into the host chromosome. After integration, phages can remain latent within the host cell as prophages until an environmental trigger leads to their excision from the genome and entry into a lytic cycle inside the cell [37]. The presence of the prophage suppresses infection by additional phages of the same strain and can sometimes even prevent infection by other phage strains [38]. This superinfection immunity is a result of the transcriptional repression of phage lytic genes [39] or, in some cases, inhibition of adsorption by additional phages [40].

In recent years, the potential importance of lysogeny in the marine environment has come to the fore and its extent and significance is under debate [37, 41, 42]. Integrated phages have been observed in many sequenced bacterial genomes [41–43] and single cells from the environment [41]. Integrase genes, used for integration, are commonly found in viromes and cellular metagenomes [42, 44, 45] and their presence is often considered to infer the ability to integrate and partake in a lysogenic lifestyle [42, 45]. Integration and excision are key processes mediating horizontal gene transfer that shape the genomes of phage and microbial populations in the oceans [37, 46], including cyanophages and cyanobacteria [27, 47, 48].

T7-like cyanophages are generally described as lytic, due to their resemblance to the lytic archetype *Escherichia coli* phage T7 and their lytic behavior during infection [5, 49, 50]. Thus, it is surprising that many T7-like cyanophages carry an integrase gene, in some cases followed by putative attachment sites (*attP*) [13, 16, 17], two lysogeny-associated features that are known to facilitate site-specific integration in lysogenic phages and are absent from T7 itself. This raises the possibility of lysogeny in the T7-like cyanophages. However, intact prophages are absent from marine unicellular *Synechococcus* and *Prochlorococcus* isolates, despite evidence for remnants of phage integration [47, 51, 52]. In addition, efforts to isolate marine *Synechococcus* or *Prochlorococcus* lysogens of any type have failed [3, 5, 52], making it unclear whether this phenomenon occurs for cyanophages in these cyanobacteria.

Here, we developed a method for genetic engineering of cyanophages that is highly effective for two distinct and dominant cyanophage families that infect marine *Synechococcus* and *Prochlorococcus*, the T7-like and T4-like cyanophages. Employing this method, we provide experimental evidence for the integration of T7-like cyanophages into their host’s genome despite a lytic infection cycle, and show that both the integrase gene and *attP* site are required for this process. We found that the frequency of integration was low both in the lab and the oceans. Stable lysogens of T7-like cyanophages were not found, however, despite repeated attempts in an experimental setup designed to obtain them even in the absence of superinfection immunity. These results suggest that lysogeny does not occur in T7-like cyanophages and that integration is less regulated and more transient than that described for classical lysogenic phages.

## Results and discussion

### REEP: a genetic engineering system for cyanophages

Genetic engineering of phages requires two stages. The first is the generation of mutations in the phage genome and the second is the isolation of the mutant phages. The genetic engineering system presented here is based on naturally high rates of homologous recombination between plasmid and phage DNA during infection. Following recombination, an enrichment procedure and a PCR screen are used for the isolation of mutant phages. We name this method REEP for REcombination, Enrichment and PCR screening. This is a simple and generalizable method particularly well suited for ecologically relevant host-virus systems for which genetic tools are limited.

### High frequency of recombination between cyanophage and plasmid DNA during infection

**Fig. 1:**
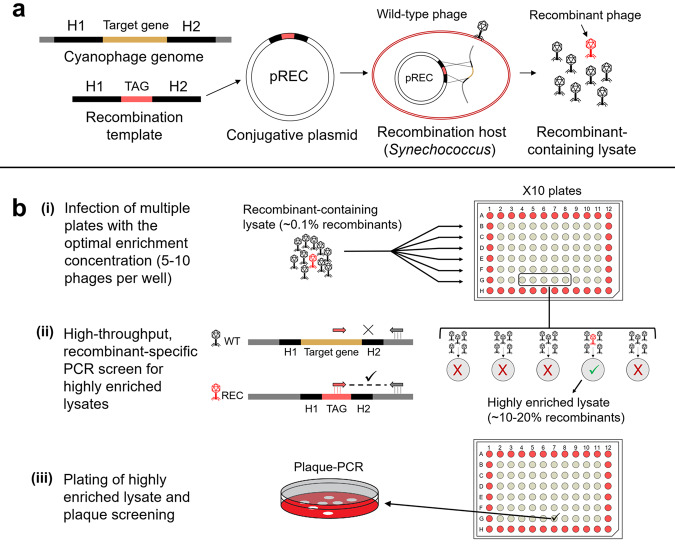
Diagram of the REEP genetic engineering system for cyanophages. **a** The process of producing recombinant-containing phage lysates. A recombination template is composed of homologous regions (H1, H2, 200–300 bp each) that flank the target gene to be deleted in the phage genome and a short TAG sequence that will be inserted in place of the target gene. This is cloned into the pREC plasmid and inserted into *Synechococcus* to produce the recombination host. This host is infected with wild-type cyanophages. Homologous recombination that occurs during infection produces a lysate containing wild-type (black) and recombinant (red) phages. **b** Enrichment and screening for the isolation of recombinant phages. (**i**) Multiple 96-well plates are infected with the recombinant-containing lysate at the optimal enrichment dilution, which is 5–10 phages per well. (**ii**) Once lysis is complete, the plates are screened by recombinant-specific PCR to detect recombinant phages based on their TAG sequence. Wells in which a recombinant phage was present among the initial 5–10 phages used for infection will now contain a lysate that is highly enriched with recombinants (>100-fold) and will be detected in the PCR screen. (**iii**) The highly enriched lysates from PCR positive wells are plated for plaque screening and isolation of the recombinant phage.

The first stage of any genetic engineering system is the generation of mutations. In the REEP method, as in various other phage genetic engineering methods [53–55], phage mutations are generated during infection by homologous recombination between cyanophage DNA and recombination templates carried on replicative plasmids inside cyanobacterial hosts (Fig. 1a). Recombination templates consist of two regions, 200–300 bp long (Table 1), that are homologous to those flanking the target gene in the phage genome. This length of homologous regions was chosen to be long enough to increase the probability for homologous recombination but without cloning an entire phage gene into the recombination template, which may be lethal when introduced into the host. A short foreign DNA TAG sequence (20–60 bp long) is positioned between the homologous regions and replaces the target gene with minimal interruption to the compact phage genome. This TAG sequence is used later for detection of recombinant phages by PCR. The recombination templates are cloned into a broad host-range, mobilizable plasmid in *E. coli* and inserted into marine *Synechococcus* by conjugation [56] to produce recombination host strains (see “Methods” for details). Infection of a recombination host with the wild-type phage results in lysates with a mixture of wild-type and recombinant phages (recombinant-containing lysates).

**Table 1:** Frequency of recombinant phages in recombinant-containing lysates for various mutations in different cyanophages.

Mutant strain name	Parent cyanophage strain	Phage type	Deleted sequence (size)	Inserted sequence (size)	Homology length (left/right)	Recombinant phage frequency (*n* = 3)
S-TIP37-Δ*int*	S-TIP37	T7-like	*gp11*- integrase (836 bp)	pRL-TAG (57 bp)	266/290 bp	8.48E-04 ± 8.61E-04
S-TIP37-Δ*attP*	S-TIP37	T7-like	*attP* - putative integration (67 bp)	DR-TAG1 (23 bp)	234/234 bp	3.71E-03 ± 3.11E-04
S-TIP37-Cm	S-TIP37	T7-like	Non-coding region downstream of *gp45* (22 bp)	proCAT (961 bp)	268/268 bp	3.61E-04 ± 2.09E-04
Syn5-Δ*int*	Syn5	T7-like	*gp17* - integrase (508 bp)	pRL-TAG (57 bp)	251/226 bp	6.75E-04 ± 1.77E-04
Syn9-*Δ6.5*	Syn9	T4-like	Non-coding region downstream of *gp6* (14 bp)	DR-TAG1 (23 bp)	251/274 bp	9.57E-04 ± 3.77E-04
					Average	1.31E-03 ± 1.22E-03

We tested this recombination system for cyanophages belonging to the two major cyanophage families and investigated loci not expected to be essential for phage reproduction. The host strain used was *Synechococcus* sp. strain WH8109. The generation of recombinant-containing phage lysates was successful for all three cyanophages tested: S-TIP37 and Syn5 from the T7-like cyanophages, and Syn9 from the T4-like cyanophages (Table 1). Recombinant phages were produced at an average frequency of 1.31 × 10^−3^ (0.1%) for five recombinant phages, ranging from 3.61 × 10^−4^ to 3.71 × 10^−3^, as determined by quantitative PCR (qPCR) (Table 1). This range of frequencies was observed for various deletion and insertion sizes and was not dependent on the family or strain of phage investigated (Table 1). These recombination frequencies were high enough to suggest that screening, rather than selection systems, could be used to isolate recombinant phages. For comparison, recombination rates reported previously for other phages range from nearly 10^−3^ [57] to as low as 10^−9^ [58].

### A generalizable method for the screening and isolation of recombinant phages

The next stage in a genetic engineering method is the isolation of recombinants. Although the recombination step described above is quite efficient, detecting recombinant phages by PCR screening thousands of individual plaques would be very time consuming and labor intensive. To overcome this problem, we developed a procedure that combines an enrichment step for recombinant phages and a high throughput PCR screening procedure for detection of recombinant-enriched lysates (Fig. 1b). In this procedure, hundreds of host cultures, grown in 96-well plates, are infected with subsamples of the recombinant-containing lysate at a concentration of 5–10 phages per well. This concentration is pre-determined using the most probable number method (MPN) and ensures lysis of all infected wells. Though most wells contain only wild-type phages, wells with a recombinant phage generate lysates that are generally enriched more than 100-fold to contain 10–20% recombinants rather than the ~0.1% recombinants present in the initial recombinant-containing lysate (Table 1).

Once lysis of the infected cultures is visible, a PCR screen is performed to detect wells enriched with recombinant phages that are identified based on the inserted TAG sequence (Fig. 1b). It is generally sufficient to screen 3–10 96-well plates (180–600 wells, infected with 900–6000 phages) to detect several wells highly enriched with recombinant phages. PCR can be performed on each plate individually or after pooling samples from each row of 10 plates into a single plate and screening the 10 individual wells that went into a positive pooled well. This reduces the number of PCR reactions tenfold, although may reduce the sensitivity of detecting recombinant phages. The contents of the PCR-positive wells are plated on cyanobacterial lawns for plaque formation and ~10–50 individual plaques are screened by PCR to obtain an isolated recombinant phage that is further purified and taken for validation (see “Methods”).

The REEP method’s power lies in its simplicity. It requires only the ability to insert a plasmid into the bacterial host and homologous recombination, which is a frequent process in many DNA and RNA viruses [59, 60]. Even for viruses where homologous recombination is less frequent, it can be enhanced by expression of a recombination system commonly used for genetic engineering (e.g., the Lambda RED system) [61]. Furthermore, this method does not require any specialized equipment or tools and the enrichment and screening is relatively high throughput when using 96-well plates and multi-channel pipettes. It generates mutants with minimal disruption to the phage genome, using a very short sequence to replace the target gene, and can potentially be used to generate scarless mutations (i.e., without insertion of a TAG, with screening done based on differences in amplicon size). The REEP method was successfully employed on all three cyanophages tested in this study and was efficient in generating a range of deletions and insertions in cyanophages belonging to two distinct phage families with different characteristics, including vast differences in genome size and content. This demonstrates the flexibility of this method, a valuable feature for studying a set of phage strains, as often done in ecologically relevant systems.

Many methods for genetic engineering of bacteriophages exist [61–63]. Although the generation of mutations is quite straightforward, a major obstacle is the isolation of the mutant phages. This is largely due to the lack of generalizable selection markers for phages, similar to those used in bacteria, such as antibiotic resistance genes. Specialized systems have been developed for well-studied model phages that often rely on strain-specific characteristics [62, 63]. For example, host genes that are essential for phage infection but not for host growth can be used as selection markers. CRISPR-Cas based methods have been proposed as a generalizable approach for counter-selection against wild-type phages [53, 64], as have the use of reporter genes to isolate recombinant phages by visual screening [54, 55]. However, setting up such systems can be difficult and time consuming, especially as they require expression of foreign genes in the host, often entailing the development and optimization of suitable regulatory elements and codon optimization. This is no trivial task when working with non-model organisms with a limited set of molecular tools and is likely to require specific adjustments for each host-virus system of interest. In addition, overexpression of a reporter gene, as well as the insertion of a large fragment into the compact phage genome, may have an effect on the phage phenotype beyond that of the deletion of the gene of interest, confounding results from infection experiments with such mutant phages.

Prior to the development of the REEP method, we examined several other approaches for isolation of recombinant phages without success (see supplementary information for details). First, three different CRISPR-Cas based methods were constructed for targeting and eliminating non-recombinant cyanophages [53, 64]. None of these systems were efficient against cyanophages, despite being adjusted for use in cyanobacteria. In parallel, we attempted to optimize several genes encoding fluorescent proteins for use as reporter genes for visual screening of recombinant phages, yet none were successful in generating a detectable signal above the autofluorescence of the cyanobacterial host. Another approach was screening for recombinant phages by plaque-hybridization [65], which led to the detection of recombinants, but did not yield viable recombinant cyanophages despite many attempts. Last, cloning of whole phage genomes into fosmid vectors and inserting them into *Synechococcus* by conjugation were attempted, failing in the step of transfer into *Synechococcus*. These unsuccessful attempts further emphasize the advantages of our straightforward method. It has no gene expression involved, is free of potentially confounding effects on mutant phage fitness and phenotype, and is unlikely to require changes to be transferred to other host-virus systems. These advantages make this method an ideal candidate for genetic engineering in viruses for which genetic methods are currently unavailable, especially for ecologically relevant host-virus systems that are not well characterized and have limited genetic tools.

### T7-like phage integration into marine cyanobacteria

Integrase genes are often detected in the genomes of cultured phages and bacteria as well as in viromes and metagenomes collected from the oceans [13, 16, 22, 37, 43]. Based on this, many marine phages are hypothesized to have the ability to integrate into the genome of their hosts and to have a lysogenic life cycle [37, 46]. With a genetic system in hand, we set out to experimentally test these hypotheses for T7-like cyanophages. This entailed assessing whether: (1) T7-like cyanophages integrate into the genome of their host; (2) the integrase gene is functional and, together with the *attP* site, mediate integration; and (3) stable integration ensues and superinfection immunity is conferred.

### Site-specific integration of T7-like cyanophages occurs during lytic infection

**Fig. 2:**
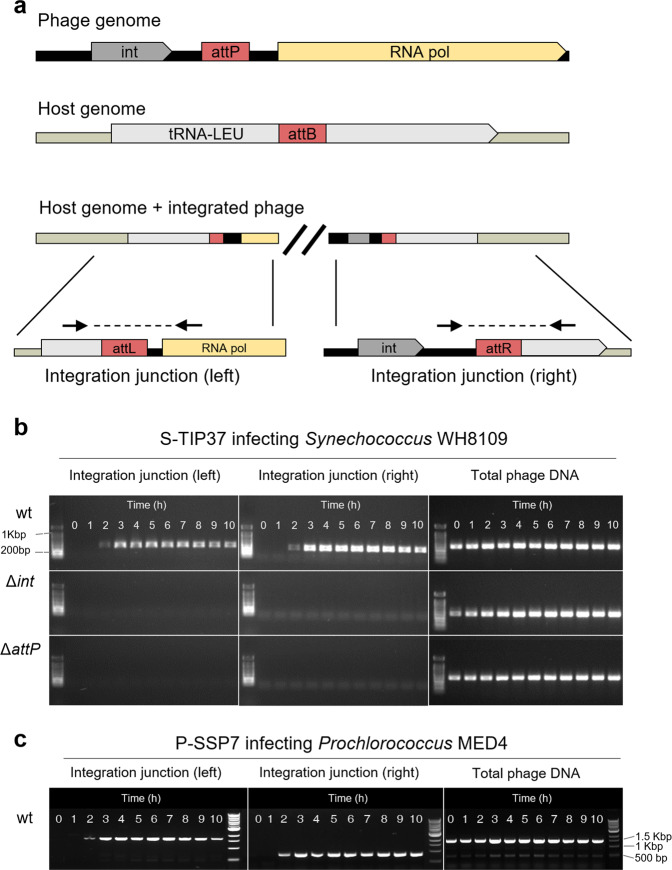
Site-specific integration of T7-like cyanophages. **a** Organization of the integration elements found in cyanophages S-TIP37 and P-SSP7, and in their host genomes. Integration junctions formed due to phage integration and the location of the primers (black arrows) used to detect them by PCR are also shown. Int- integrase, attP/B – attachment sites on the phage/bacterial genomes, respectively, attL/attR – left and right attachment sites after integration, respectively. Primers for total phage DNA assessment are located elsewhere on the phage genome (see Table S5). **b** Evidence for phage integration from a PCR assay for detection of integrated phages during infection of *Synechococcus* WH8109 with S-TIP37 phage strains: wild-type (wt), integrase deleted (*Δint*) and *attP* deleted (*ΔattP*) strains. **c** Integration of wild-type phage P-SSP7 (wt) during infection of *Prochlorococcus* MED4. All results shown here are representative of 3 independent experiments.

The presence of an integrase gene and a putative phage attachment site (*attP*) in T7-like cyanophages was first reported for the P-SSP7 phage that infects *Prochlorococcus* sp. strain MED4 [13]. The potential *attP* site is downstream of the integrase gene and is identical to a region in a tRNA-Leu gene of the host. Similarly, we found an integrase gene and a comparable putative *attP* site in the genome of S-TIP37 (Fig. 2a), a T7-like cyanophage that infects *Synechococcus* sp. strain WH8109 [66].

We first tested the ability of these two wild-type phages to integrate into the genomes of their hosts over a 10-hour period after phage addition (Fig. 2b, c). Intracellular genomic DNA was subjected to PCR assays designed to amplify across expected integration junctions when the phages integrate into the cyanobacterial tRNA-Leu gene. Both the S-TIP37 and the P-SSP7 phages were found to integrate into their hosts’ genomes at the expected site from 2 hours after infection onwards (Fig. 2b, c).

We then tested whether the integrase gene and the *attP* sites are functional and are involved in the integration process. This is particularly important because marine cyanobacteria, including both *Synechococcus* WH8109 and *Prochlorococcus* MED4, code for integrase genes [47, 67], and host enzymes have been shown to mediate phage integration in other systems [68]. We generated two mutant strains of S-TIP37 in which either the integrase gene (∆*int*) or the *attP* site (∆*attP*) were knocked out. Neither left nor right integration junctions were formed during infection with these mutant S-TIP37 phages (Fig. 2b). These results indicate that both the integrase gene and the *attP* site are essential for integration.

Interestingly, infection by both wild-type and integrase-mutant S-TIP37 phages exhibited infection dynamics and phage progeny production typical of classic lytic phages (Fig. S1). The length of the latent period and phage fitness were no different in the mutant and wild-type S-TIP37 phages (Fig. S1, S2a). Nutrient availability has previously been proposed to affect the frequency of lysogeny [37, 69, 70]. We therefore hypothesized that fewer phages would be produced (i.e., lower fitness would result) if more cells were lysogenized under nutrient deprivation. However, even when the host was grown in nutrient-depleted medium, wild-type and integration mutants demonstrated similar progeny production (Fig. S2).

**Fig. 3:**
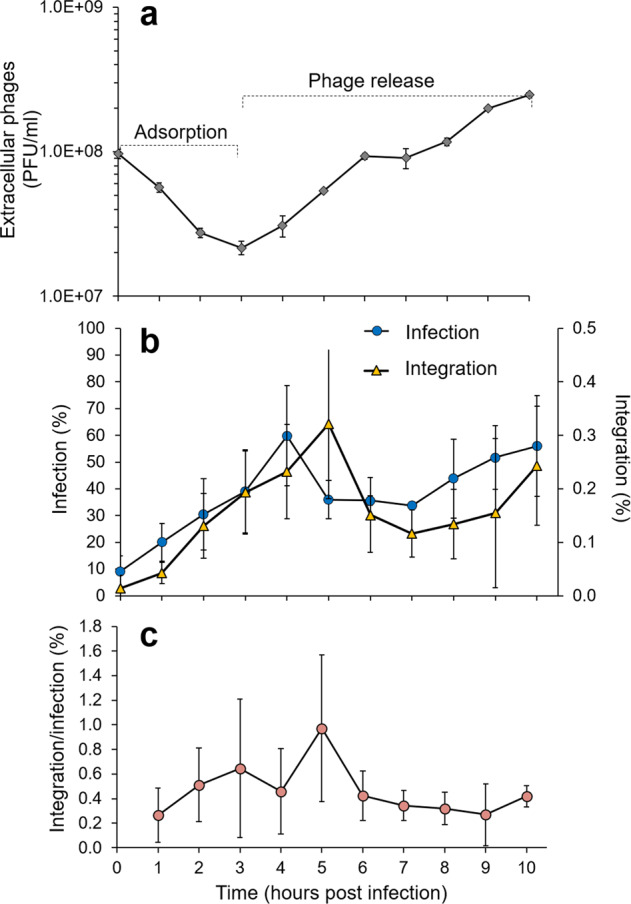
Dynamics of S-TIP37 infection and integration. **a** Phage growth curve of the wild-type S-TIP37 phage during infection of *Synechococcus* WH8109. **b** Dynamics of infected cells (blue circles, left Y-axis) and of cells containing integrated wild-type phages (yellow triangles, right Y-axis; only left junctions were tested), determined by single-cell, solid phase PCR. Percent infection and integration were calculated from the number of polonies divided by the number of cells added to the slide. **c** Average ratio of cells with integrated phages to total number of infected cells, calculated by dividing average % integration by average % infection from each timepoint. Integration at timepoint zero was below the limit of detection and was thus not used to calculate the integration/infection ratio. No integration was detected with the S-TIP37 integrase mutant, which served as a negative control in this experiment.

The above results raised the question of whether phage integration is a basic part of the lytic infection cycle, occurring in every infected cell. To assess this we quantified the number of infected cells containing an integrated phage using the iPolony method, a method capable of detecting single molecules of viral DNA in infected cells [71]. The percentage of integrated phages correlated with the number of infected cells, increasing as more phages adsorbed and entered the cell and decreasing as phages were released and cells lysed (Fig. 3). However, phage integration occurred only in a small fraction of cells, ranging from 0.04 to 0.32%, even though up to 60% of the cells were infected (Fig. 3b). Thus, the frequency of integrated phages was low, ranging from 0.3–1% of infected cells for most of the infection cycle (Fig. 3c). These findings indicate that integration is a relatively rare event, and may be transient, rather than a fundamental part of lytic infection in these cyanophages.

### S-TIP37 integration is not part of a classic lysogenic cycle

Hallmark features of lysogeny include stable integration into the host genome, a regulatory system that controls the lytic/lysogenic life cycles and superinfection immunity [37, 38, 72]. To test whether integrated T7-like cyanophages possess these features, we first searched for homologs of repressors of lytic infection present in known lysogenic phages. No such genes were identified in T7-like cyanophages. Next, we performed a series of experiments to test whether stable integration of S-TIP37 occurs when infecting its *Synechococcus* WH8109 host. Classic lysogens (e.g., Lambda lysogens of *E. coli*) can be isolated based on their superinfection immunity, by simply plating an infected culture on agar plates and testing the colonies that grow for the presence of a prophage [73]. However, when we attempted this previously for various marine cyanobacteria and their integrase-containing cyanophages (including P-SSP7 and S-TIP37), none of the resistant colonies were found to contain a prophage [6, 74]. We therefore predicted that if stable integration occurs it is extremely rare. To overcome this we used our genetic system to generate a strain of S-TIP37 carrying a chloramphenicol resistance gene (*proCAT*) in a non-coding region of the phage genome. This engineered phage strain, S-TIP37-Cm (Table 1), was allowed to adsorb to *Synechococcus* WH8109 cultures which were then plated on chloramphenicol-containing agar plates. If stable lysogens that provide superinfection immunity exist then chloramphenicol resistant colonies are expected to grow. This approach allows testing a vast number of phage-infected cells because it eliminates colonies that originate from non-infected cells as well as from spontaneous phage resistant mutants, allowing only prophage-containing colonies that express the chloramphenicol resistance gene to grow. Roughly 3 × 10^9^ cells were plated in five different experiments, which was expected to yield up to 9 × 10^5^ colonies considering the integration frequency of ~0.3% found in the previous experiment (Fig. 3b). However, no chloramphenicol resistant colonies were found. Note that the *proCAT* gene provides chloramphenicol resistance in our pREC replicating plasmids (Fig. 1a) and when expressed from the chromosome of *Synechococcus* strains [75]. This suggests that despite phages integrating into the genome of their host this integration is not stable or that superinfection immunity is not provided by the integrated phage.

We then asked whether stable integration occurs but superinfection immunity is not provided, i.e. that prophage-containing cells are killed as a result of subsequent infections. To address this question, we repeated the above experiments in a manner that removes and separates free phages from host cells, preventing a second round of infection (see “Methods”). Here too, no colonies grew on chloramphenicol plates, despite growth of colonies in the absence of chloramphenicol, which confirmed that a second round of infection and lysis was indeed prevented. Finally, to rule out the possibility that prophages exist in these latter colonies but did not provide resistance to chloramphenicol, we tested 2000 colonies for the presence of prophages by PCR and found none. These findings indicate that stable integration did not occur, irrespective of whether superinfection immunity ensues or not. We note that these experiments do not enable us to ascertain whether superinfection immunity is provided for the short period during which phages are integrated into the host genome.

Our combined findings suggest that the site-specific integration observed here in T7-like cyanophages is not a part of a classical lysogenic lifestyle, but rather is a transient process. This intrinsic lack of stable prophage integration may be due to the absence of regulatory genes that, in well studied lysogen systems, suppress the expression of lytic cycle genes and maintain the phage genome integrated into the host genome [38, 72]. Their absence can result in spontaneous excision of the integrated phage and induction of the lytic cycle, or in lysis by a superinfecting phage. In addition, lytic infection may be initiated by the integrated phage from within the host genome. Such a phenomenon was recently reported in the classic lysogenic phage Lambda, with lytic infection observed from a phage integrated into the host genome, possibly due to low expression of the CII gene coding for the repressor of the lytic cycle [76].

### T7-like cyanophage integration in the oceans

**Fig. 4:**
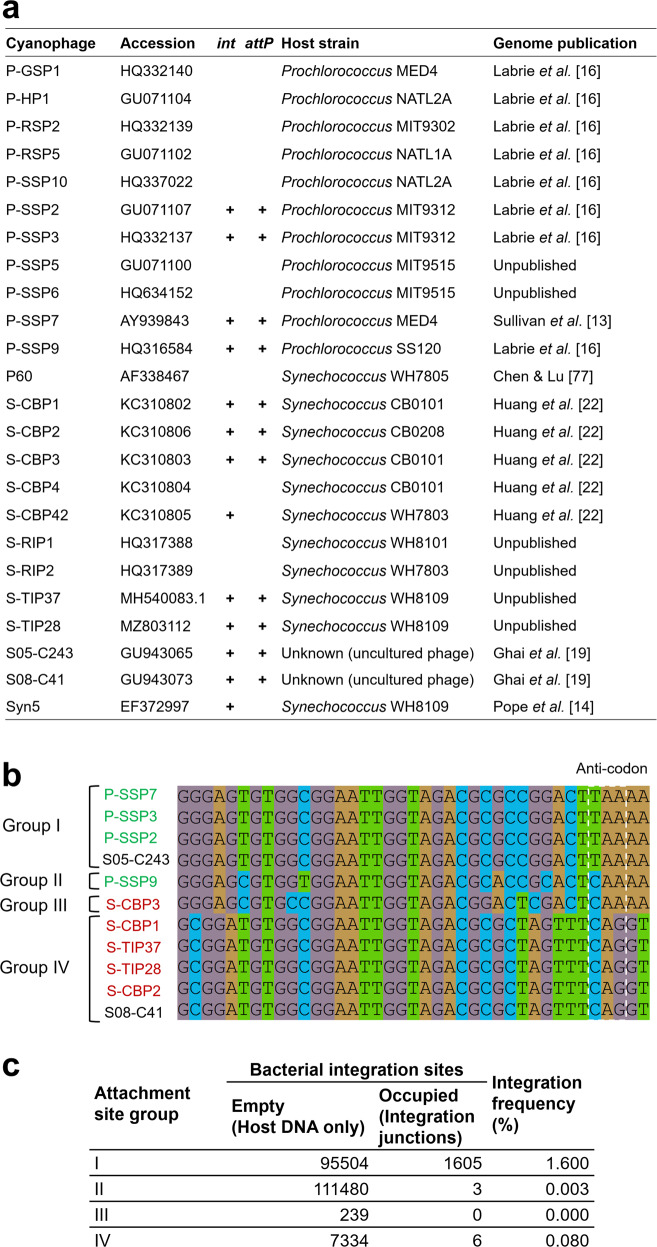
Lysogeny- associated elements present in T7-like cyanophage genomes. **a** Analysis of available T7-like cyanophage genomes for the presence of integrase genes (*int*) and *attP* sites. **b** Sequence comparison and group division of the *attP* sites found in the analyzed genome. Cyanobacterial host genus is indicated by font color of the phage name (green- *Prochlorococcus*, red- *Synechococcus*, black- unknown). **c** Frequency of integration found for cyanophages in the different attachment site groups in oceanic samples.

We sought to estimate the frequency of integrated T7-like cyanophages in cyanobacteria in the oceans. First, we searched for *attP* sites in 24 publicly available genomes in this group of cyanophages [14, 16, 19, 22, 77] by searching for homologous sequences downstream of integrase-containing cyanophage genomes in cyanobacteria (Table S1). We found that of the 13 cyanophage genomes with integrase genes, 11 also carried an *attP* site (Fig. 4). Consistent with the *attP* sites for P-SSP7 [13] and S-TIP37 (this study), all *attP* sites were perfect matches for regions in cyanobacterial tRNA-Leu genes, which can thus be considered a general cyanobacterial attachment (*attB*) site. Based on their sequences, we divided the *attP* sites into four groups, with groups I and II belonging to phages that infect *Prochlorococcus* and groups III and IV belonging to phages that infect *Synechococcus* (Fig. 4b). Second, we analyzed publicly available metagenomic datasets from six different projects (Table S2), searching for reads of cyanobacterial origin that contain these T7-like cyanophage attachment sites. The number of reads containing “empty” integration sites (i.e., cyanobacterial DNA only) or phage-host integration junctions were used to calculate the integration frequency. For the two dominant groups, I and IV, an integration frequency of 1.6% and 0.08% was found, respectively, whereas group II and III showed only 0.003% and 0% integration, respectively (Fig. 4c). Thus, similar to lab findings, T7-like cyanophage integration is rare but detectable in the oceans.

Cell density has been hypothesized to impact lysogeny. However, this is still under debate due to conflicting evidence (summarized by Brum et al. [78]). Nonetheless, our combined findings of rare integration at high cell densities in lab experiments (5 × 10^7^ cells·ml^−1^) as well as at the considerably lower cyanobacterial densities found in the oceans (ranging from ~10^4^ to 3 × 10^5^ cells·ml^−1^ [1]) argues against density being a factor for the T7-like cyanophages.

## Conclusions

The discovery of phages as highly abundant and diverse members of the community in virtually every habitat on Earth [9, 10, 79, 80] has fueled recognition of their ecological importance. Over the past two decades there has been an explosion in the sequence information available for environmental viruses. Yet a substantial portion of viral genomes contains genes of unknown function [16, 17, 22, 30, 36], as well as other genes with putative functions that were unexpected in viral genomes, leading to intriguing hypotheses as to their role in infection [13–15]. The lack of a genetic system broadly applicable to ecologically relevant models has precluded gaining fundamental understanding of the many novel features of these significant members of the natural world. This method now provides the opportunity, in the post-genomics era, to assess the function of interesting virus genes directly in the infection process and their impact on virus fitness and evolution. Thus, the REEP genetic engineering method developed here places marine cyanophages among the few model organisms that are culturable, genetically engineerable and ecologically significant.

The possibility of integration and a potential lysogenic lifestyle in T7-like phages was first hypothesized 15 years ago for T7-like cyanophages [13, 14, 16, 22] and more recently for T7-like pelagiphages [81, 82], based on the presence of integrase genes and *attP* sites in their genomes. In our first use of the REEP method we addressed this hypothesis for the T7-like cyanophages. Our findings unequivocally indicate that these elements are used for integration into the host genome even though these are essentially lytic phages. Indeed, no evidence for stable integration nor lysogeny was found, although we cannot categorically rule it out for all members of this family or under all environmentally relevant conditions. Nonetheless, the absence of intact prophages in cyanobacterial genomes, including hundreds of single-cell genomes from diverse environments [83, 84], provides independent evidence for the lack of classical lysogeny in these phages. Furthermore, lysogeny-derived immunity was never the mechanism of resistance among hundreds of experimentally selected resistant cyanobacterial strains, including against the P-SSP7 [6] and S-TIP37 [74] phages, even though integration occurs at a 1000-fold greater frequency than the appearance of resistance mutations [6]. Thus, although the presence of the integrase gene facilitated integration when an *attP* site was also present, we caution against automatically equating the presence of the gene with a lysogenic lifestyle.

The physiological or evolutionary benefit that marine T7-like cyanophages obtain from carrying these elements and transiently integrating into host genomes remains uncertain. One feasible scenario is that integration provides a safety net for phages that cannot complete their lytic cycle. This may be important when environmental conditions are not suitable for lytic infection. Alternatively, this process may allow defective phages to be rescued through complementation or recombination with other phages infecting the same host cell, and perhaps even facilitate the formation of new genetic combinations, accelerating phage evolution. Clearly, phage integration is important for host and phage evolution through horizontal gene transfer [37, 46]. Transient integration in up to 1% of infections may enhance the frequency of the introduction of new genes into microbial hosts as well as the capture of microbial genes by viruses in the environment.

## Materials and methods

### Culture growth

Cultures of *Synechococcus* sp. strain WH8109 were grown in artificial seawater (ASW) medium [85], with modifications as described elsewhere [86], at 21 °C and a light intensity of 30 µmol photons m^−2^ s^−1^. *Prochlorococcus* sp. strain MED4 was grown in Pro99 medium [87], at 18 °C and 70 µmol photons m^−2^ s^−1^. All cultures were grown under a 14:10 light-dark cycle. Chlorophyll *a* fluorescence (excitation at 440 nm, emission at 660 nm) was used as a proxy for cell density and was measured in 96-well plates using a Synergy Mx Microplate Reader (Biotek). Pour-plating was performed as previously described [56], using the ASW growth medium with low-melting point agarose at a final concentration of 0.28% [87, 88] and the addition of 1 mM sodium sulfite [88]. For isolation of cyanobacterial colonies, a heterotrophic helper strain, *Alteromonas* sp. EZ55, was added to the pour-plate mixture [89]. All bacterial and phage strains used in this work are listed in Table S3. Experimentation was carried out at cell abundances of ~3–5 × 10^7^ cells/ml for *Synechococcus* WH8109 and *Prochlorococcus* MED4.

### Lysate preparation and phage growth curves

Lysates were prepared by infecting cyanobacterial cultures at early logarithmic phase with phage from either a single plaque or a pre-existing liquid lysate. Once full lysis of the culture was observed, lysates were filtered through 0.22 µm syringe filters (Millex-GV, Millipore) and stored in glass tubes at 4 °C. Plaque assays were used to determine infective phage concentrations. Lysate samples were diluted and plated in agarose plates containing cyanobacteria at concentrations sufficient to produce lawns, allowing formation of plaques. Phage growth curve experiments were performed by infecting cyanobacterial cultures at a multiplicity of infection (MOI) of 1-3, with subsamples collected over a period of 10 h. To measure the concentration of infective phages in the extracellular medium samples of 0.1 ml were collected, added to 0.9 ml of medium, and filtered through a 0.22 µm syringe filter to remove cyanobacterial cells and the filtrate used in plaque assays.

### Construction of plasmids and bacterial conjugation

The plasmid pDS-proCAT (Fig. S3) was used as a backbone for the construction of all recombination plasmids used in this work. This replicative plasmid is a derivative of the broad host-range mobilizable vector pRL1342 (received as a gift from Peter Wolk [90]). It was modified by replacing the original chloramphenicol resistance gene with a cyanobacterial-optimized copy, *proCAT*. This gene was codon-optimized for expression in several *Prochlorococcus* strains and was found to work well in *Synechococcus* strains. For efficient expression, the *proCAT* gene was fused to the *rnpB* promoter and the *atpB* ribosome-binding site of *Prochlorococcus* MED4. Recombination templates were constructed either by the PCR overlap extension method [91] or by a two-step cloning procedure. A short TAG sequence (20–60 bp) was inserted between two regions of homology on the phage genome (~250 bp long) (H1 and H2) that flank the region to be deleted (Fig. 1a). Recombination plasmids (pREC) were inserted by electroporation into *E. coli* strain DH10b, an efficient recipient of large plasmids. Plasmids were extracted from PCR-positive colonies using a miniprep kit (NucleoSpin Plasmid EasyPure, MACHEREY-NAGEL) and transformed into *E. coli* SM10 or S17.1, conjugation donor strains carrying the *λpir* gene [92]. Conjugation of plasmids into *Synechococcus* was done as described previously [56] and resulting recombination hosts were grown in the presence of 1 μg/ml chloramphenicol. Plasmids used in this study are listed in Table S4.

### Production of recombinant-containing cyanophage lysates and estimating recombinant frequencies

Recombination plasmids were conjugated into *Synechococcus* sp. strain WH8109 to form recombination hosts. Recombination hosts were infected with wild-type phages at a low MOI (~0.01). After lysis of the cultures, lysates were filtered and the 0.2 µm filtrate was stored as described above. The presence of recombinant cyanophages was tested by PCR, using primers that anneal to the TAG sequence and specifically detect recombinants (Table S5) using 2x Taq PCR mix (Tiangen) and 2 μl of the lysate (in 20 μl reactions). Recombinant phage frequency was determined for several representative lysates by real-time qPCR (see below).

### Isolation of recombinant cyanophages by enrichment and PCR screening

Recombinant phages were enriched in 96-well plates containing cultures of *Synechococcus* sp. strain WH8109 at early logarithmic growth phase. The optimal phage enrichment concentration was determined by the MPN method. *Synechococcus* cultures grown in a 96-well plate were infected with 10-fold serial dilutions of the recombinant-containing lysate, with each dilution used to infect a single row of wells. The optimal enrichment concentration is operationally determined to be the highest tenfold serial dilution at which lysis is observed in an entire row of wells. This concentration is typically 5–10 phages per well, and is used in the subsequent enrichment step to infect the internal 60 wells of three to ten 96-well plates containing *Synechococcus* cultures. Once the cultures lyse, the wells were screened by PCR for the presence of recombinant phages in 20 µl reactions using 10 µl Taq PCR MasterMix (Tiangen), 0.4 µM primers and 2 µl of phage lysate (without prior filtration). Samples were run on 1.5% agarose gels with ~0.2 µg·ml^−1^ ethidium bromide in large electrophoresis trays containing 200 wells each. Lysates from PCR-positive wells were filtered through a 0.22 µm syringe filter and plated. Plaques were purified twice and verified by PCR to obtain pure recombinant phage mutants. Full genome sequencing of all phage strains (Table S3), including the wild-type parental strains, were performed to verify the mutation and to ensure an identical genetic background to the wild-type phage.

### Real-time quantitative PCR to measure recombination frequencies

Recombination frequencies were determined by real-time qPCR, in which the copy-number of recombinant and non-recombinant phage DNA was quantified. DNA templates were extracted from phage lysates using Wizard PCR Preps DNA Purification Resin and Minicolumns (Promega), as previously described [93]. Template DNA and primers (0.2 µM each) were added to LightCycler 480 SYBR Green I Master mix (Roche) and amplified using a LightCycler 480 Real-Time PCR System (Roche). The LightCycler 480 software was used to calculate the number of cycles required to reach the optimal fluorescence threshold (Ct). All amplicons tested by qPCR were first amplified by PCR and cloned into plasmids using the TOPO TA Cloning Kit (Thermo Fischer). Plasmid DNA carrying the tested amplicons was extracted and used to generate calibration curves for calculation of the absolute DNA copy number in each qPCR reaction.

### Detection of integrated phages using PCR

Cells were collected over the course of phage growth curve experiments for PCR detection of phage DNA integrated into the cyanobacterial genome. Samples were filtered onto polycarbonate 0.22 µm pore-size membrane filters (GE), washed twice with growth medium to remove free phages, and washed once with 3 ml of preservation solution (10 mM Tris, 100 mM EDTA, 0.5 M NaCl, pH 8) [94, 95]. The filter was flash-frozen in liquid nitrogen and stored at −80 °C. A heat lysis method was used to extract DNA from cells collected on filters [94, 95]. Filters were resuspended in 10 mM Tris-HCL solution (pH = 8), shaken in a bead-beater (Mini-BeadBeater, Biospec) for 2 min (3450 oscillations/min) without beads. Samples were heated at 95 °C for 15 minutes and the supernatant collected. For detection of integrated phage DNA, 2 µl of intracellular template DNA were used in 20 µl PCRs (Taq PCR MasterMix, Tiangen), as described above. Primers specific for detection of total phage DNA and integration junctions were used (Table S5).

### Quantification of infected cells and integrated phages using the iPolony method

For single-cell quantification of integrated phages, cultures of the *Synechococcus* WH8109 host were infected with the wild-type S-TIP37 phage (MOI = 3). The S-TIP37 integrase mutant was used as a negative control in infection experiments with the host strain. Samples from phage-infected cultures were collected at different time points, fixed in 0.1% glutaraldehyde, incubated for 20 min in darkness, flash-frozen in liquid nitrogen and stored at −80 °C. Samples were sorted using an Influx flow cytometer (BD) based on their chlorophyll *a* fluorescence (excitation at 488 nm, emission at 580/30 nm) and forward scatter to remove free phages and excess glutaraldehyde [71]. A known number of cells (quantified using the same flow cytometer) were added to iPolony reactions designed for intracellular DNA detection [71]. Briefly, a mixture of cells, PCR reagents and polyacrylamide gel were poured into custom-made glass slides (11.6 µl final volume). A 15 min cell lysis step was followed by in-gel PCR in a slide thermal cycler (DNA Engine with dual-block slide chamber, Bio-Rad). An acrydite-modified primer (Table S5) covalently binds one PCR strand to the gel, creating local DNA amplification clusters, termed polonies (PCR-colonies) [96]. Polonies were hybridized with a Cy5-modified fluorescently labeled DNA probe (Table S5) after removal of the unbound PCR strand. Hybridized polonies were identified using a GenePix 4000B microarray scanner (Axon Instruments). Plasmids harboring the tested amplicons (with left integration junction or total phage) served as positive controls for PCR amplification and efficiency. Percent integration was adjusted for the efficiency of detection of single virus genome copies which was 33% for S-TIP37 [71].

### Testing for stable integration and superinfection immunity

The S-TIP37-Cm strain was constructed by inserting the *proCAT* chloramphenicol resistance gene into the non-coding region between *g45* and *g46* of the S-TIP37 phage. This engineered phage strain was used to infect *Synechococcus* WH8109 cultures at a high MOI of 3. After a 2 h incubation to allow phage adsorption, 10^8^ cells per plate were plated in the presence of 1 μg·ml^−1^ chloramphenicol. A total of 3 × 10^9^ cells was plated in 5 independent experiments.

To differentiate between lack of stable integration and lack of superinfection immunity, in the event of no chloramphenicol resistant colonies, we carried out the above experiment after physically preventing subsequent infection of cells by free phages. Cells were diluted to 10^5^ cells·ml^−1^ and sorted to remove free phages [71] remaining after the 2 h adsorption step. In addition, cells were plated at various dilutions with and without chloramphenicol to physically distance potential prophage-containing cells from phages newly released by lytically infected cells. A total of 15 plates were plated at each dilution in 3 independent experiments. The presence of colonies in the absence of chloramphenicol indicated that this procedure prevented infection by remaining or newly released phages. The efficiency of plating of these phage-exposed cultures was approximately one-third of the non-infected control cultures, as expected from the percent infection measured two hours post infection (see Results and Discussion). Over 2000 of these colonies, that grew in the absence of chloramphenicol and originated either from non-infected cells or from lysogens, were tested for the presence of prophage by PCR.

### Fitness assays

To assess the fitness of the different phage strains, *Synechococcus* cultures were infected over a 2-day period beginning with a low MOI of 0.001. Phage abundances were determined by plaque assay at the beginning and end of experiment. A 2-day period was used to ensure host cultures did not become a limiting resource for phage production [97]. Phage fitness, expressed as the number of population doublings per day, was calculated as (log_2_(*N*_*t*_/*N*_0_))/*t*, where *t* is the assay time in days and N_0_ and Nt are the phage concentrations at the beginning and end of the experiment, respectively [97, 98]. Growth of the host culture was measured by chlorophyll *a* fluorescence (see above). For growth under nutrient deprivation (Fig. S2), a low-nutrient ASW medium was used, containing all ingredients but at 10% nutrient concentrations for NaNO_3_, NaH_2_PO_4_, NaHCO_3_ and trace metals. Exponentially growing *Synechococcus* cultures were centrifuged for 5 min at 5200 RCF, and resuspended in this low nutrient ASW medium. Nutrient deprivation was verified by reduced chlorophyll *a* fluorescence relative to growth in 100% ASW medium.

### Analyses of *attP* sites and estimation of cyanophage integration frequencies in the oceans

To identify potential attachment sites between cyanophages and their putative hosts we compared 727 *Prochlorococcus* and *Synechococcus* genomes (Table S1) against 12 integrase-carrying cyanophage genomes (Fig. 4) using BLAST (BLAST v2.6.0 + : blastn -task blastn -reward 1 -penalty −4 -gapopen 5 -gapextend 2 -perc_identity 94 -evalue 10e-5). We filtered hits of 39-44 bp length, collapsed those overlapping by at least 20 bp, and only kept the ones present in more than 10 host genomes and directly downstream of the integrase gene (bedtools v2.27.1) [99]. From the curated alignment of the corresponding sequences, we identified four distinct 39 bp attachment site motifs, all of which match the first half of a tRNA-Leu gene.

To estimate how many cyanobacteria in the wild carry integrated phages, we screened 1093 publicly available metagenomic libraries from six different marine sequencing projects [100–104] (Table S2) for reads containing a match to one of the four attachment site motifs with up to two differences (BBMap v38.16: bbduk.sh k = 39 edist = 2) [105]. We determined the locations of putative attachment sites in the cyanobacterial host genomes by BLAST best hit against the four attachment site motifs with a minimum bit score of 52. Based on those data, we generated a database of fragments containing the attachment site motif and 500 bp of up- and downstream flanking regions for all combinations of cyanobacteria and phages containing the same site. We then mapped the pre-screened metagenomic reads to this database using bwa mem v0.7.16a-r1181 and filtered the results using a custom Perl script (alignment length ≥ 100 bp, aligned read fraction ≥95%, overlap with flanking regions ≥ 20 bp) [106, 107]. Finally, we obtained counts for “empty” and “occupied” integration sites by counting reads matching to “host-att-host” and “host-att-phage”/“phage-att-host” fragments using a custom R script.

## Supplementary information

Supplementary text and figures

Supplementary tables
