# Supplementary material for: Genetic engineering of marine cyanophages reveals integration but not lysogeny in T7-like cyanophages
Source: ISME J. 2021 Aug 24;16(2):488–99. doi: 10.1038/s41396-021-01085-8 (PMC8776855; doi:10.1038/s41396-021-01085-8)
Supplement: Supplementary file 1 — Supplementary text and figures [file 41396_2021_1085_MOESM1_ESM.pdf]

Genetic engineering of marine cyanophages reveals integration but not lysogeny in T7-like cyanophages

Dror Shitrit[1], Thomas Hackl[2], Raphael Laurenceau[2], Nicolas Raho[2], Michael C.G. Carlson[1], Gazalah Sabehi[1], Daniel A. Schwartz[1], Sallie W. Chisholm[2] and Debbie Lindell[1*]

[1]Faculty of Biology, Technion – Israel Institute of Technology, Haifa, Israel, 3200003.
[2]Department of Civil and Environmental Engineering, Department of Biology, Massachusetts Institute of Technology, Cambridge, MA, USA, 02142.

Supplementary information

Supplementary text 2

Fig. S1: Infection dynamics comparison of S-TIP37 strains 4

Fig. S2: Fitness assessment of S-TIP37 strains under different growth conditions 5

Fig. S3: The pDS-proCAT plasmid map 6

Dataset file:

Table S1: Cyanobacterial genomes used for phage attachment site (*attP*) analyses

Table S2: Metagenomic databases used in this study

Table S3: Strains used in this study

Table S4: Plasmids used in this study

Table S5: Oligonucleotides used in this study

Supplementary text

Additional genetic engineering methods attempted to generate mutant cyanophages

Prior to the development of the REEP method described in this study, we attempted several other approaches based on previously described methods and principles (see review by Pires *et al.* [1]). The first approach was to use CRISPR-Cas based methods for selection of recombinant phages, an approach previously used in other phage-host systems [2,3]. We constructed and tested three such selection systems. The first was based on *Streptococcus pyogenes* Cas9 combined with its native crRNA [4]. The second employed a codon-optimized gene encoding the *S. pyogenes* Cas9, combined with guide RNA's that replaced the crRNA [5]. The third system used the *Francisella novicida* Cpf1 nuclease and guide-RNA [6]. In the latter two systems, the nuclease genes and guide-RNAs were fused to cyanobacterial promoters. Despite testing numerous targets in different cyanophages, no phage interference was observed.

We also attempted to use reporter genes to replace the target region on the phage genome and to isolate the mutants by a visual screen of the plaques. For this, we constructed a collection of genetic cassettes in which genes encoding fluorescent proteins were fused to regulatory sequences designed for efficient expression in cyanobacteria. The reporter genes included mCherry and mOrange [7], Venus [8] and eGFP [9], as well as codon-optimized super-folder GFP [10], mScarlet and mTagBFP2 [11]. All of these genes were fused to the cyanobacterial *rnpB* promoter and *atpB* ribosome binding site, as used in the *proCAT* gene cassette. They were then cloned into the pDS-proCAT plasmid and strains of *Synechococcus* WH8109 harboring these plasmids were generated. The fluorescence of these strains was tested by a fluorometer (Synergy Mx Microplate Reader, Biotek), a fluorescent microscope (model TE2000-E; Nikon) and visually, by using suitable light filters and a long exposure camera. However, none of these strains produced significant fluorescence, despite sufficient mRNA levels that were detected by RT-qPCR. This may be due to interference from inherent autofluorescence of the *Synechococcus* host, or a result of insufficient expression at the protein level.

Another approach we tried to use was screening for recombinant phages by plaque hybridization [12]. For this, the recombinant-containing lysate was plated on bacterial lawns for plaque formation. The plaques were transferred onto a nylon membrane and hybridized with a probe targeting the TAG sequence. Although the presence of recombinant plaques was detected by the probe, we were unable to obtain viable recombinant phages, despite repeated attempts.

Lastly, cloning of whole phage genomes into fosmid vectors was also attempted as a method to engineer phage genomes. High molecular weight DNA of S-TIP37 was purified and successfully cloned into the pEpiFOS-5 vector using the EpiFOS fosmid library production kit (Epicentere). An origin of transfer (*oriT*) site was cloned into the fosmid to make it mobilizable into *Synechococcus* WH8109. The conjugated cells were plated with wild-type *Synechococcus* WH8109 to allow plaque formation. However, no plaques were formed. It is important to note that such fosmids would not be able to accommodate T4-like cyanophages as their genomes are much larger than the 45-50 kb cargo limit of the fosmids.

References

1. Pires DP, Cleto S, Sillankorva S, Azeredo J, Lu TK. Genetically engineered phages: a review of advances over the last decade. Microbiol Mol Biol Rev. 2016;80(3):523–43.

2. Kiro R, Shitrit D, Qimron U. Efficient engineering of a bacteriophage genome using the type I-E CRISPR-Cas system. RNA Biol. 2014;11(1):42–4.

3. Martel B, Moineau S. CRISPR-Cas: An efficient tool for genome engineering of virulent bacteriophages. Nucleic Acids Res. 2014;42(14):9504–13.

4. Jiang W, Bikard D, Cox D, Zhang F, Marraffini LA. RNA-guided editing of bacterial genomes using CRISPR-Cas systems. Nat Biotechnol. 2013;31:233–9.

5. Cong L, Ran FA, Cox D, Lin S, Barretto R, Habib N, et al. Multiplex genome engineering using CRISPR/Cas systems. Science. 2013;339(6121):819823.

6. Ungerer J, Pakrasi HB. Cpf1 is a versatile tool for CRISPR genome editing across diverse species of cyanobacteria. Sci Rep. 2016;6:39681.

7. Shaner NC, Campbell RE, Steinbach PA, Giepmans BNG, Palmer AE, Tsien RY. Improved monomeric red, orange and yellow fluorescent proteins derived from *Discosoma* sp. red fluorescent protein. Nat Biotechnol. 2004;22(12):1567–72.

8. Nagai T, Ibata K, Park ES, Kubota M, Mikoshiba K, Miyawaki A. A variant of yellow fluorescent protein with fast and efficient maturation for cell-biological applications. Nat Biotechnol 2002 201. 2002;20(1):87–90.

9. Cormack BP, Valdivia RH, Falkow S. FACS-optimized mutants of the green fluorescent protein (GFP). In: Gene. Elsevier B.V.; 1996. p. 33–8.

10. Kremers GJ, Goedhart J, Van Den Heuvel DJ, Gerritsen HC, Gadella TWJ. Improved green and blue fluorescent proteins for expression in bacteria and mammalian cells. Biochemistry. 2007;46(12):3775–83.

11. Subach OM, Cranfill PJ, Davidson MW, Verkhusha V V. An enhanced monomeric blue fluorescent protein with the high chemical stability of the chromophore. PLoS One. 2011;6(12):28674.

12. Dale JW, Greenaway PJ. Identification of recombinant phages by plaque hybridization. In: Walker JM, editor. Nucleic Acids. Totowa, NJ: Humana Press; 1984. p. 285–8.

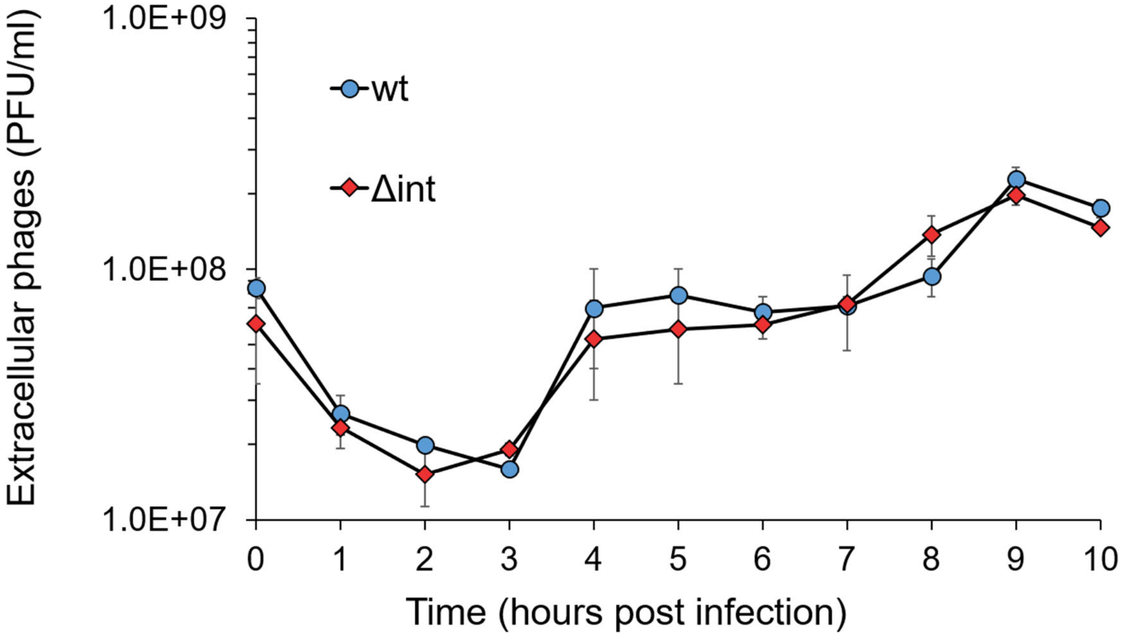

Fig S1. Infection dynamics of S-TIP37 strains. Growth curves of wild-type S-TIP37 (wt) and the integrase mutant (Δint). Average and standard deviation of three biological replicates. Note that the length of the latent period and the lytic cycle for the mutant phage is the same as in the wild-type phage.

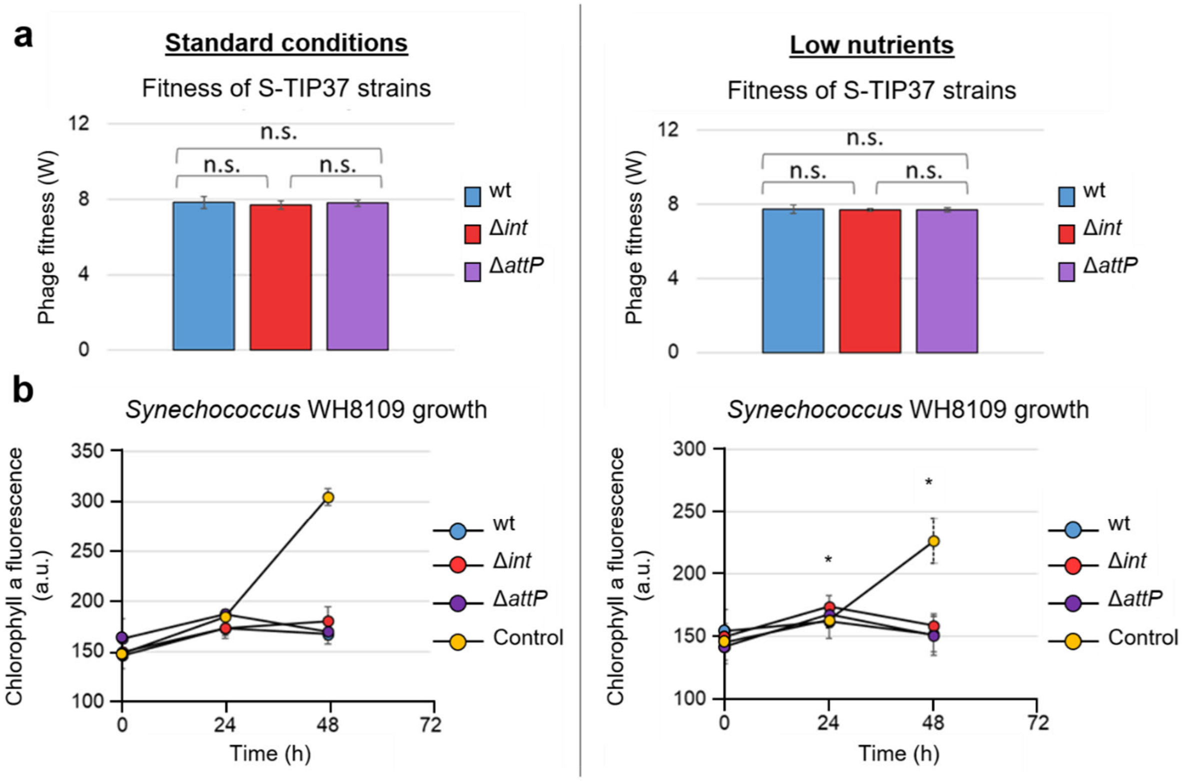

Fig. S2. Fitness assessment of S-TIP37 strains under different growth conditions. a. Phage fitness shown as the number of doublings per day (W) of the wild-type (wt), Δ*int* and Δ*attP* strains of S-TIP37, infecting exponentially growing *Synechococcus* transferred to standard (left) and low (right) nutrient medium. Non-significant differences (two-tailed T-Test, n=3) are marked as "n.s.". **b.** Culture growth of the host during infection with the three different phage strains (wt, Δ*int*, Δ*attP*) and without phage infection (control). Significant differences in chlorophyll *a* fluorescence of the control cultures between standard and low nutrient conditions are marked (*$p<0.05$, two-tailed T-Test, n=3). The lower chlorophyll *a* fluorescence in response to nutrient deprivation could be due to either reduced growth of the culture or reduced levels of pigmentation relative to the culture grown in the standard medium.

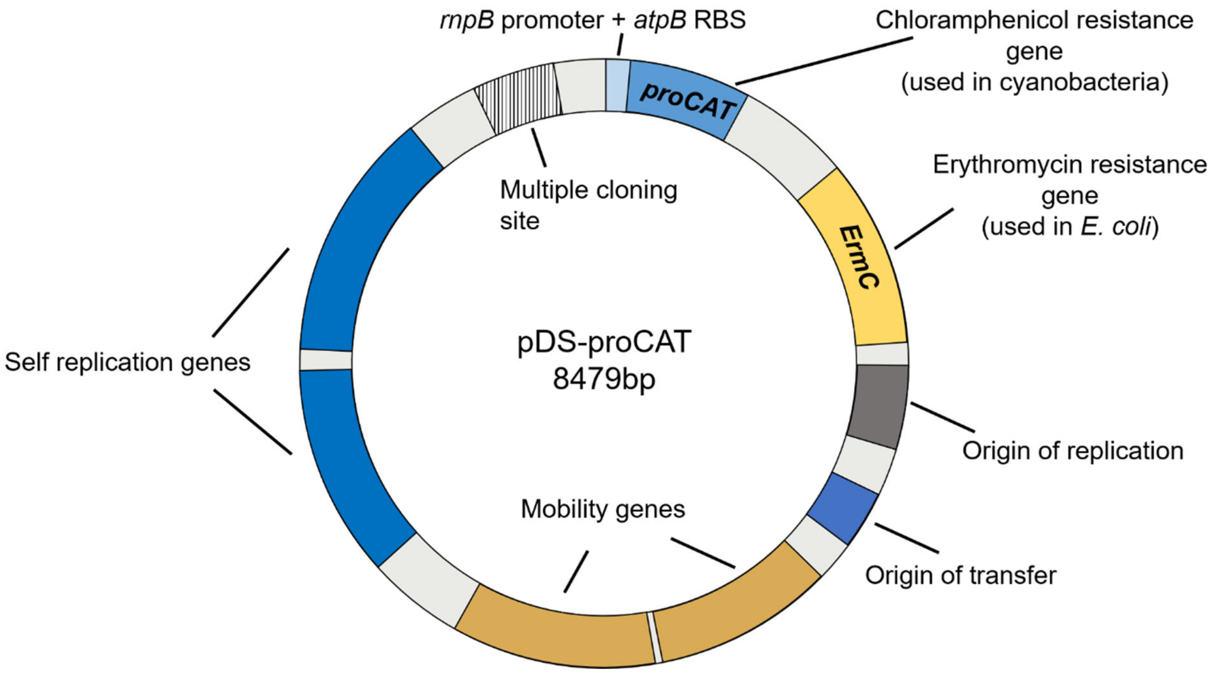

Fig S3. The pDS-proCAT plasmid map
